# “Salvage techniques” are the key to overcome difficult biliary cannulation in endoscopic retrograde cholangiopancreatography

**DOI:** 10.1038/s41598-022-17809-5

**Published:** 2022-08-10

**Authors:** Shikiko Maruta, Harutoshi Sugiyama, Sadahisa Ogasawara, Chihei Sugihara, Mayu Ouchi, Motoyasu Kan, Toshihito Yamada, Yoshifumi Miura, Hiroki Nagashima, Koji Takahashi, Yuko Kusakabe, Hiroshi Ohyama, Koichiro Okitsu, Izumi Ohno, Rintaro Mikata, Yuji Sakai, Toshio Tsuyuguchi, Jun Kato, Naoya Kato

**Affiliations:** 1 Department of Gastroenterology, Graduate School of Medicine, Chiba University, Inohana 1-8-1, Chiba-City, 260-8670 Japan; 2 Translational Research and Development Center, Chiba University Hospital, Chiba, Japan; 3 The Chiba Prefectural Sawara Hospital, Chiba, Japan

**Keywords:** Gastroenterology, Bile ducts

## Abstract

Although the efficacy and safety of salvage techniques for biliary cannulation in endoscopic retrograde cholangiopancreatography (ERCP) have been reported, few reports analyzed the choice of techniques and their clinical outcomes in large cohorts. This study aimed to evaluate the outcomes of biliary cannulation in patients with native papillae. We retrospectively identified 1021 patients who underwent initial ERCP from January 2013 to March 2020. We investigated background factors, treatment details, cannulation success rates, and adverse event rates. Then we analyzed a series of treatment processes, including salvage techniques such as double guidewire technique (DGT), needle knife pre-cutting (NKP), and transpancreatic pre-cut papillotomy (TPPP). The initial ERCP success rate using standard technique alone was 62.8%, which increased to 94.3% including salvage techniques. Salvage techniques were frequently required in patients with long oral protrusions (OR 2.38; 95% CI 1.80–3.15; p < 0.001). A total of 503 cases (49.3%) had long oral protrusions, 47.5% of which required the salvage techniques, much higher than 27.5% of not-long cases. Patients with long oral protrusions had a higher frequency of NKP. In conclusion, patients with long oral protrusions frequently required salvage techniques. Salvage techniques may help to overcome many difficult biliary cannulation cases.

## Introduction

Since 1968, endoscopic retrograde cholangiopancreatography (ERCP) has helped diagnose and treat biliary and pancreatic diseases^1,2^. For over five decades, it has been an established endoscopic procedure through the innovation in equipment and the improvement of techniques by endoscopists. Previously, percutaneous transhepatic biliary drainage (PTBD) was the commonly used technique to approach the bile duct. However, nowadays, PTBD has been replaced by ERCP in countries where endoscopic techniques are widely available due to the high invasiveness and the low quality of life for patients of PTBD^3,4^.

Selective biliary cannulation is one of the basic techniques of ERCP. However, the rate of unsuccessful biliary cannulation is approximately 5–20% in the native papilla because of difficult cases, even with skilled experts^5–8^. Most studies have defined a difficult biliary cannulation according to a minimum number of cannulation attempts or the time taken to cannulate, but the consensus has not been completely agreed to. Increasing the number of attempts for biliary cannulation and the long procedure time leads to the risk of post-ERCP pancreatitis (PEP)^9^. Therefore, completing biliary cannulation reliably for difficult cases is one of the most important clinical issues of the ERCP procedure.

To date, various salvage techniques have been developed for difficult biliary cannulation cases in ERCP. Two well-known and typical salvage techniques are the double guidewire technique (DGT) and the pre-cut technique^10,11^. DGT can be performed in the case of unintentional pancreatic guidewire insertion. The guidewire is kept in the pancreatic duct, and cannulation of the bile duct is attempted with a second guidewire. On the other hand, pre-cut techniques can be classified into two main types: needle knife pre-cutting (NKP) and transpancreatic pre-cut papillotomy (TPPP). NKP is freehand pre-cut starting either from the papillary orifice or the ampulla. TPPP is the pancreatic guidewire-assisted method, which can be performed over the pancreatic wire with a standard sphincterotome to expose the biliary orifice.

A variety of salvage techniques have been almost standardized. Several studies reported success rates and adverse events of each technique, although the analysis of all reports extracted only cases in which each salvage technique was performed^5,11–20^. On the other hand, several articles have reported the factors that make biliary cannulation difficult in ERCP^21–25^. However, few of them have described how difficult cases were addressed using salvage techniques. An analysis of difficult cases of biliary cannulation for all patients who underwent ERCP, including selecting salvage techniques and their clinical outcomes, would clarify the whole procedure stream to select the best salvage techniques in real-world practice. To the best of our knowledge, no comprehensive analysis of approaching bile ducts in ERCP with clinical outcomes of salvage techniques for difficult cases has been reported. Therefore, this study aimed to evaluate the clinical outcomes of ERCP in whole clinical courses from reaching major papilla until achieving biliary cannulation in patients receiving ERCP with native papillae. Focusing on salvage techniques, we also conducted a detailed analysis of the technique selection when biliary cannulation was unsuccessful by the standard approach.

## Methods

### Study design

This was a single-center retrospective cohort study carried out in a tertiary referral hospital in Japan. The research ethics committee of the Graduate School of Medicine, Chiba University, approved this study (Approval number 4036) and waived informed consent. All methods were carried out in accordance with relevant guidelines and regulations.

### Patient population

From January 2013 to March 2020, 5332 ERCPs were performed at Chiba University Hospital. A total of 1153 patients had native papillae and indications for selective biliary cannulation. The patient exclusion criteria were: (1) surgically altered anatomy (Billroth II gastrectomy or Roux-en-Y anastomosis), (2) unreachable papillae, (3) invisible papillae within a large diverticulum, (4) age under 20 years, (5) determined to be inappropriate (cases with missing reports or images for some reason, or cases biliary cannulation was performed through a fistula instead of a papilla). After excluding patients who met at least one of the exclusion criteria, we analyzed the data of 1021 patients (Supplementary Fig. S1).

### Endoscopic procedures

Midazolam and pentazocine were injected for sedation, in doses depending on age and tolerance, with continuous monitoring of the pulse rate, respiratory rate, oxygen saturation, and blood pressure. ERCP was performed using a standard iodinated contrast medium, with a side-viewing therapeutic duodenoscope (JF-260V, TJF-260V, or TJF-Q290V; Olympus, Tokyo, Japan). Endoscopic sphincterotomy was performed using a sphincterotome (CleverCut3V; Olympus) with a diathermy generator (ERBE VIO3 or VIO300D; Tubingen, Germany) by applying a mixed current in the “endo cut” mode.

All endoscopists were classified as experts or trainees. Each “expert” had at least three years’ experience in the specialized pancreaticobiliary team at the tertiary referral center, had performed over 300 ERCP-related procedures per year, and could achieve selective biliary cannulation in more than 90% of cases. Six experts and 20 trainees performed ERCPs as main endoscopists over the study period of approximately seven years.

### Cannulation strategy

In most cases, biliary cannulation was attempted using a cannula (PR-104Q; Olympus) with 0.025-inch guidewires (mostly Olympus Visiglide2, and in a few cases, pre-modified Visiglide or PIOLAX Revowave) loaded. We first approached with the contrast-assisted method, and switched to the guidewire-assisted method when it was not easy. We defined “Failed biliary cannulation after 10 min or repeated unintentional pancreatic access (≥ 3 times)” as one criterion of difficult biliary cannulation, and decided to switch from trainee to expert or to shift to salvage techniques (described later). In cases of repeated unintentional pancreatic duct approach or when guidewire placement would increase the stability of the procedure, we attempted to insert the guidewire into the pancreatic duct with minimal contrast guidance. We routinely measured time, keeping in mind that prolonged procedures would be a risk factor for adverse events such as pancreatitis.

### Salvage techniques

We defined salvage technique as any technique other than the standard technique using contrast-assisted or guidewire-assisted methods. The three main techniques were DGT, TPPP, and NKP.

#### DGT

After placement of the guidewire in the pancreatic duct, the cannula containing a second guidewire was passed into the same working channel of the scope alongside the other guidewire. Biliary cannulation was attempted first in the region toward the upper left of the pancreatic duct orifice where the guidewire was placed.

#### TPPP

**Figure 1:**
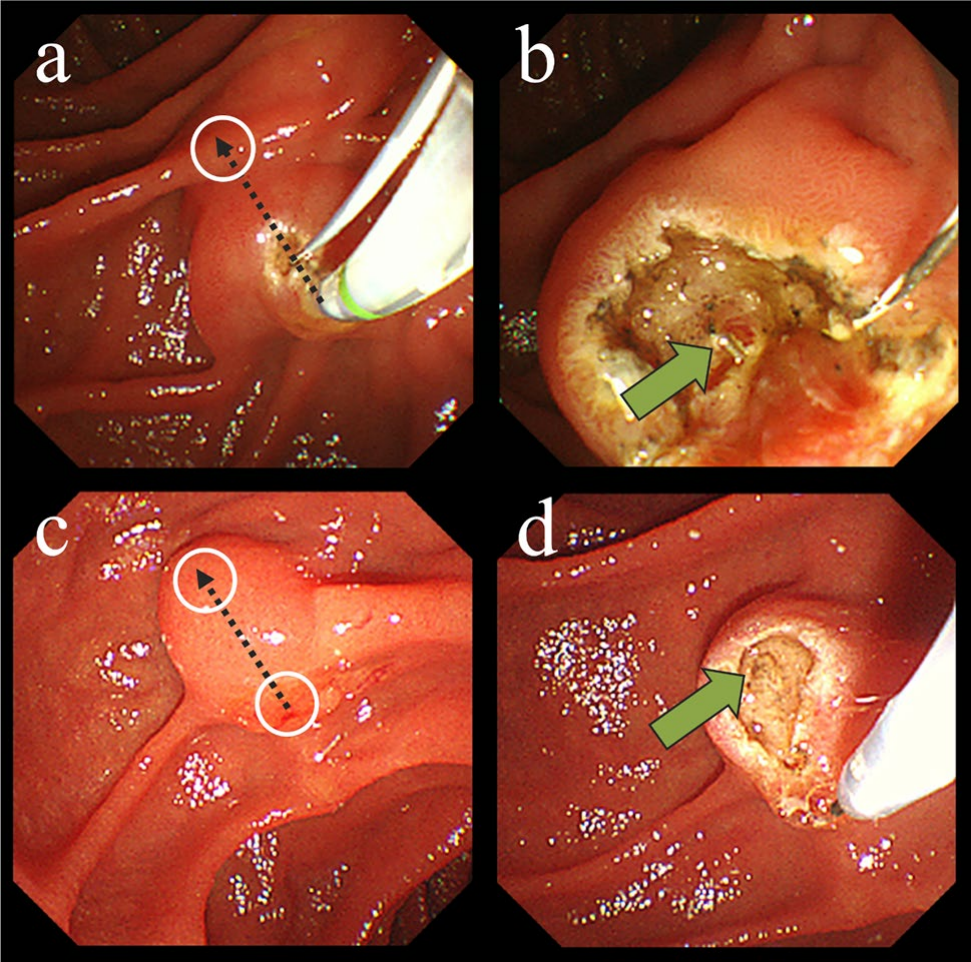
Endoscopic images of TPPP and NKP. (**a**) TPPP. A guidewire was placed in the pancreatic duct, and then a regular sphincterotome was wedged into the pancreatic orifice. The incision was made in the 12 o’clock direction (black arrow). The incision was first made up to the hooding folds (white circle), and then additional incisions were made as needed to expose the lumen of the common bile duct. (**b**) After TPPP. The orifice was cut wide enough to expose the lumen of the common bile duct (green arrow). (**c**) NKP. The needle was partially extended beyond the tip of the catheter. Then, an incision was performed toward the intraduodenal segment of the bile duct in the 12 o’clock direction (black arrow), starting at the papillary orifice (white circle at the starting point of the arrow). The incision was first made up to the most elevated point of the ampulla (white circle at the end of the arrow), and then additional incisions were made as needed to expose the lumen of the common bile duct. (**d**) After NKP. The papilla was cut wide enough to expose the lumen of the common bile duct (green arrow). *TPPP* transpancreatic pre-cut papillotomy, *NKP* needle knife pre-cut.

After placement of the guidewire in the pancreatic duct, a regular sphincterotome was wedged into the pancreatic orifice. The incision length was decided until the bile duct can be well exposed. Thus, TPPP was performed until the incision would be made up to the point where the hooding folds were cut off (In some cases, the incision may be made until it reaches the midpoint of the oral protrusion). A sufficient incision was intentionally made to expose the lumen of the common bile duct. After that, we attempted biliary cannulation with the same cannula containing a second guidewire based on the partially exposed bile duct mucosa or bile outflow as a indicator (Fig. 1a,b).

#### NKP

Using a single-lumen needle knife sphincterotomes (KD-10Q-1 or KD-V441M; Olympus), NKP was performed in cases without pancreatic duct approach while attempting standard techniques or in cases with difficult guidewire placement due to inflection or stenosis of duct. A wide incision was made from the papillary orifice to expose the lumen of the common bile duct (Fig. 1c,d). We attempted biliary cannulation with a cannula, preceded by a guidewire in most cases. In the case of needle knife fistulotomy (NKF), the starting point of the incision was the ampulla. When the orifice itself was difficult to confront well, NKF was selected.

When a guidewire was placed in the pancreatic duct, the choice between DGT and TPPP depended on the case and the endoscopist’s experience. TPPP was often performed immediately after pancreatic guidewire placement when the expert was the main endoscopist or in cases with poor scope stability expected to make DGT difficult. On the other hand, DGT was often performed first when the trainee was the main endoscopist or especially in cases with a high risk of bleeding. Since our institution is a teaching facility, trainees often assigned the main endoscopist. When biliary cannulation with DGT was not successful, TPPP was then considered in order to achieve biliary cannulation according to the judgment of an expert (main endoscopist or supervisor) and the patient’s tolerance for the procedure. Most DGT attempts were limited to less than 3 min and three times with a view to early pre-cut transition in our institution.

Whether or not selective biliary cannulation was successful, most patients with pancreatic guidewire placement underwent pancreatic duct stenting to prevent PEP. A 5-Fr, 3-cm externally flanged straight prophylactic pancreatic duct stent (Wilson-Cook Medical, Winston-Salem, North Carolina, USA) was used. In addition, NSAIDs suppositories and aggressive hydration were used in high-risk cases, such as young women and patients with a history of pancreatitis.

### Definitions and classifications

In this study, cannulation time was calculated from the time of frontal view of Vater’s papilla to the time when deep biliary cannulation was achieved with the cannula or the guidewire on fluoroscopy. Total operation time was calculated from the time of scope insertion into the esophagus to scope removal.

The oral protrusion pattern was classified into two types, according to the length of the oral protrusion. We defined “Long” as more than double the ratio of the length of the oral protrusion to the transverse diameter of the papilla.

Adverse events of ERCP were classified and graded according to consensus guidelines^26^. The diagnostic criteria for PEP were abdominal pain lasting > 24 h after ERCP and hyperamylasemia (> 3 times the upper limit of the normal range).

### Statistical analysis

Pearson’s chi-squared test was used to compare proportions between categorical variables. Two-sample t-tests were used to compare continuous variables. P-values of < 0.05 were considered statistically significant. According to univariate analysis, factors with a significant effect on the requirement for salvage techniques were further analyzed by multivariate analysis using logistic regression. Odds ratios and 95% confidence intervals were calculated. The data were analyzed using JMP Pro 15 (SAS Institute, Japan).

## Results

### Patient characteristics

This study included 1021 patients (634 males, 387 females; median age, 70 years [IQR 64–77]). Table 1 lists indications for ERCP, anatomical and morphological factors of Vater’s papilla or duodenum, and site of bile duct stricture. Bile duct stones, biliary cancer, and pancreatic cancer were the most common indications. Malignant diseases accounted for more than half of the total number of cases.

**Table 1:** Characteristics of 1021 patients underwent ERCP with native papilla.

	All analyzed patients (n = 1021)
**Age―median (IQR)**	70 (64–77)
**Male**	634 (62.1%)
**Indications for ERCP**	
Benign	448 (43.9%)
Bile duct stone	337 (33.0%)
Benign biliary stricture	96 (9.4%)
Malignancy	573 (56.1%)
Biliary cancer	246 (24.1%)
Pancreatic cancer	188 (18.4%)
Lymph node and liver metastasis of any cancer	83 (8.1%)
Hepatocellular carcinoma	56 (5.5%)
Papillary cancer	23 (2.3%)
**Long oral protrusion**	503 (49.3%)
**Peripapillary diverticulum**	202 (19.8%)
**Tumor invasion to the papilla or oral protrusion**	56 (5.5%)
**Distal biliary stricture**	357 (35.0%)
**Main pancreatic duct stricture**	182 (17.8%)

*IQR* interquartile range, *ERCP* endoscopic retrograde cholangiopancreatography.

### Success rate and adverse event rate

**Figure 2:**
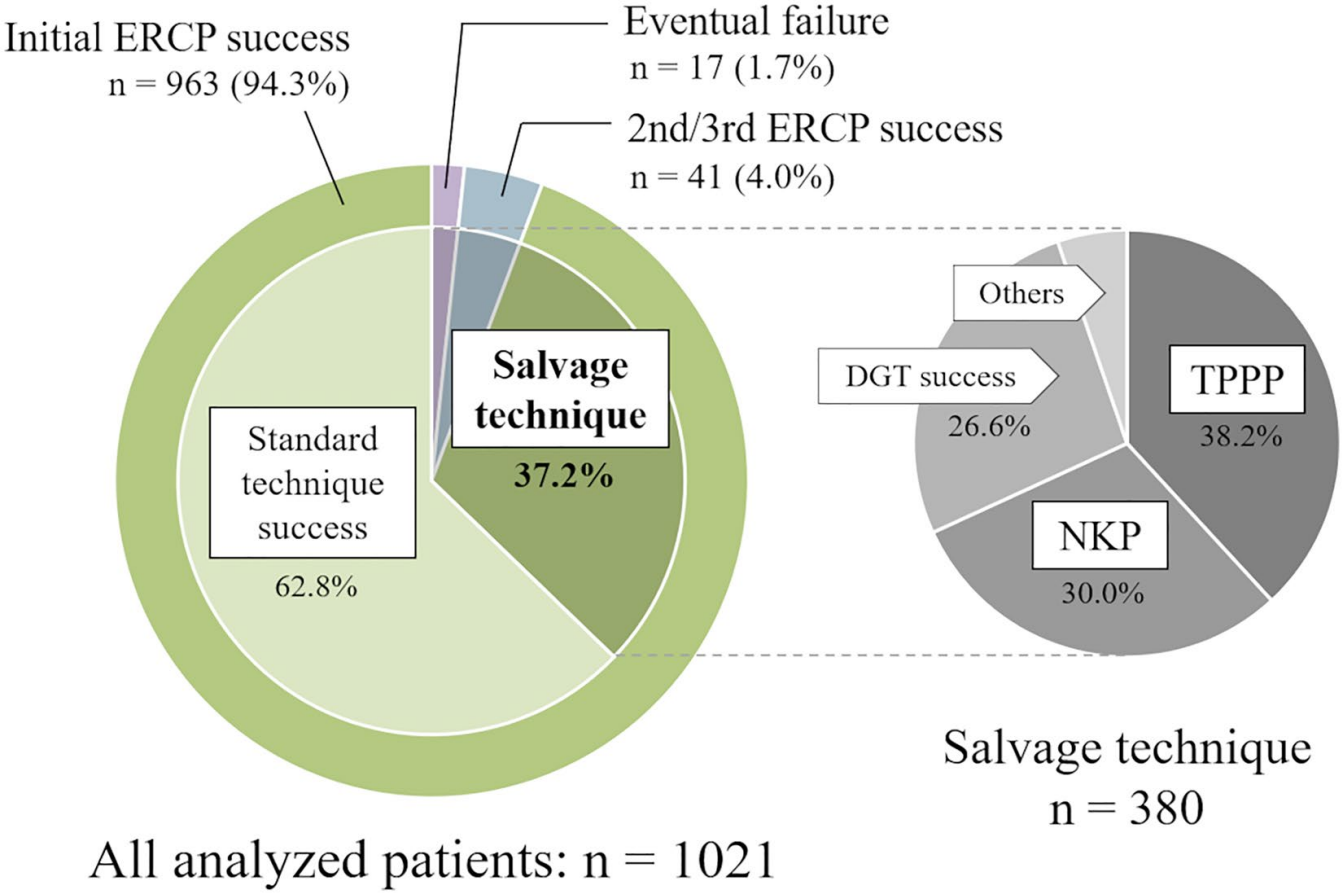
Frequency of salvage techniques required and success rate of cannulation. Thirty-seven percent of all cases required salvage techniques and the components was not significantly biased. *ERCP* endoscopic retrograde cholangiopancreatography, *NKP* needle knife pre-cut, *TPPP* transpancreatic pre-cut papillotomy, *DGT* double-guidewire technique.

The success rate of biliary cannulation including salvage techniques in initial ERCP was 94.3% (n = 963), and the eventual success rate, including 2nd and 3rd ERCPs, was 98.3% (n = 1004) (Fig. 2). Initial ERCP was unsuccessful in 58 patients, and 44 of those patients undertook ERCP again. Forty-one of them were eventually successful, and 6 of them required additional incisions after pre-cut.

PEP was observed in 46 patients (4.5%). Bleeding occurred in 33 patients (3.2%), with during the procedure in 15 patients (1.5%) and post-procedure in 21 patients (2.1%). Cholangitis was observed in 12 patients (1.2%), and no perforation was observed. The median cannulation time was 8 min (IQR 3–18), and the median total operation time was 36 min (IQR 26–49). Prophylactic pancreatic stents were used in 236 patients, corresponding to 95.2% of the 248 patients with pancreatic guidewire placement.

### Cases that required salvage techniques

**Figure 3:**
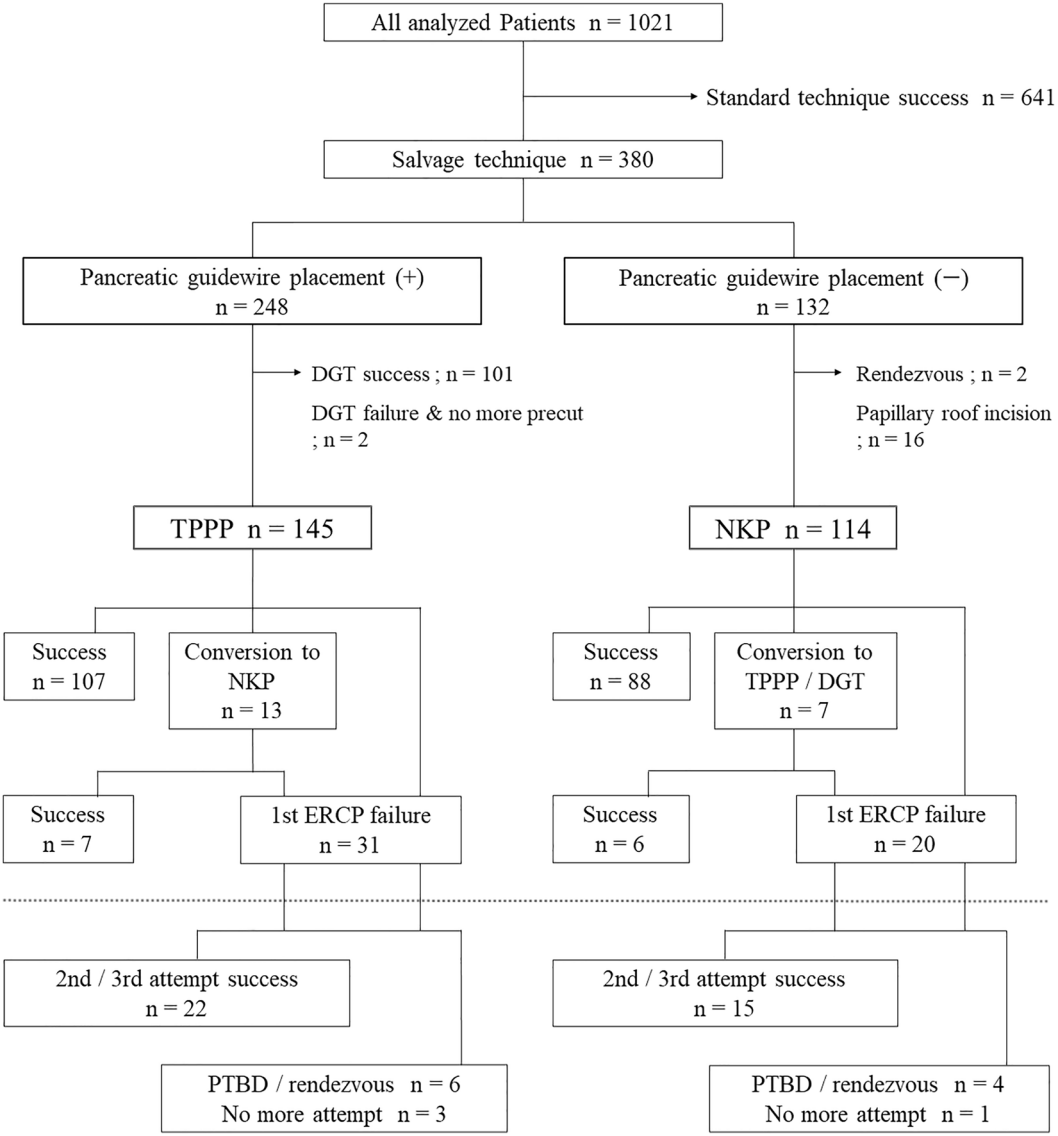
A flowchart showing the biliary cannulation process in 1021 cases. This figure shows what techniques were applied successfully or unsuccessfully in the 380 cases that required salvage techniques, and if unsuccessful, what approaches were used next. In this study, biliary cannulation using the rendezvous technique was counted as an unsuccessful ERCP. *ERCP* endoscopic retrograde cholangiopancreatography, *NKP* needle knife pre-cut, *TPPP* transpancreatic pre-cut papillotomy, *DGT* double-guidewire technique, *PTBD* percutaneous transhepatic biliary drainage.

Figure 3 is a flowchart showing the biliary cannulation process in 1021 cases. Salvage techniques were required in 380 patients (37.2%). Guidewire was placed in the pancreatic duct while attempting biliary cannulation in 248 patients, and 179 (72.2%) of them were applied DGT. The success rate of biliary cannulation by DGT alone was 56.4% (n = 101), and TPPP was performed in 145 patients, including those with unsuccessful DGT, while NKP was performed in 114 patients. The success rate of the initial ERCP without conversion therapy (i.e., the addition of the other pre-cut technique) was 73.8% (n = 107) in the TPPP group and 77.2% (n = 88) in the NKP group. Including conversion therapy, the success rates were 78.6% (n = 114) in the TPPP group and 82.5% (n = 94) in the NKP group, and the eventual success rates were 93.8% (n = 136) in the TPPP group and 95.6% (n = 109) in the NKP group. There was no significant difference between the TPPP and NKP groups in any comparison (Table 2). The number of successful cannulations with either the TPPP or NKP pre-cut technique was 245, which was approximately a quarter of the total number of successes (Supplementary Fig. S2).

**Table 2:** Comparison of outcomes between TPPP and NKP groups.

	TPPP (n = 145)	NKP (n = 114)	P value
Initial success by either TPPP or NKP	107 (73.8%)	88 (77.2%)	0.529
Initial success including conversion therapy	114 (78.6%)	94 (82.5%)	0.441
Eventual success	136 (93.8%)	109 (95.6%)	0.520
Cannulation time (min)―median (IQR)	29 (23–36)	29 (24.5–35)	0.899
Total operation time (min)―median (IQR)	51 (44–61.5)	50 (40.5–60.5)	0.469
Pancreatitis	16 (11.0%)	5 (4.4%)	0.066
Bleeding	10 (6.9%)	6 (5.3%)	0.585

*TPPP* transpancreatic pre-cut papillotomy, *NKP* needle-knife pre-cutting, *IQR* interquartile range.

When 69 patients without DGT try and 76 patients with unsuccessful DGT were studied separately in the TPPP group, only biliary cannulation time was significantly longer in patients with unsuccessful DGT (32.6 ± 11.3 min vs. 28.1 ± 9.7 min, p = 0.027), but there was no significant difference in cannulation success rate or adverse event rate. In the TPPP group, 125 patients (86.2%) underwent prophylactic pancreatic stenting. The incidence of hyperamylasemia was significantly higher in the TPPP group (n = 33, 22.8%) than in the NKP group (n = 14, 12.3%); however, there was no significant difference in pancreatitis. There was no significant difference in the incidence of bleeding between the TPPP and NKP groups, too.

No specific factors were detected when comparing successful and unsuccessful cases in the TPPP group. On the other hand, in the NKP group, pancreatic cancer with head pancreatic duct stricture was significantly more common in unsuccessful cases (12 [60.0%] vs. 23 [24.5%], p = 0.002). Bleeding was significantly more frequent in unsuccessful cases in both the TPPP and NKP groups (8 [25.8%] vs. 2 [1.8%] in the TPPP group [p < 0.001], 4 [20.0%] vs. 2 [2.1%] in the NKP group [p = 0.009]). Pancreatitis was significantly more frequent in unsuccessful cases in the TPPP group (8 [25.8%] vs. 8 [7.0%], p = 0.003), but there was no significant difference in the NKP group (5 [5.3%] vs. none, p = 0.585). Of the 114 patients in the NKP group, 19 were treated with NKF and 95 were treated with NKPP, and there were no significant differences in success and adverse event rates.

In the salvage technique group (patients who required the salvage techniques), there were significantly more cases of age > 70 (p = 0.005), pancreatic cancer (p < 0.001), long oral protrusion (p < 0.001), tumor invasion to the papilla or oral protrusion (p = 0.004), distal biliary stricture (p = 0.014), and head pancreatic duct stricture (p < 0.001) than those in the standard technique group (Table 3). In multivariate analysis, age > 70 (OR 1.54; 95% CI 1.18–2.01; p = 0.002) and long oral protrusion (OR 2.38; 95% CI 1.80–3.15; p < 0.001) were significantly associated with the requirement for salvage techniques. In contrast, bile duct stones, both symptomatic (OR 0.51; 95% CI 0.34–0.77; p < 0.001) and asymptomatic (OR 0.64; 95% CI 0.42–0.97; p < 0.001), were associated with success in the standard techniques.

**Table 3:** Multivariable logistic regression analysis, assessing independent predictors of salvage technique for biliary cannulation.

	Standard technique (n = 641)	Salvage technique (n = 380)	Univariate analysis (P value)	Multivariate analysis	
				Odds ratio for salvage technique (95% CI)	P value
**Age > 70**	296 (46.2%)	210 (55.3%)	0.005	1.54 (1.18–2.01)	0.002
**Indications for ERCP**					
Symptomatic bile duct stone	127 (19.8%)	49 (12.9%)	0.005	0.51 (0.34–0.77)	0.001
Asymptomatic bile duct stone	113 (17.6%)	48 (12.6%)	0.034	0.64 (0.42–0.97)	0.034
Other benign disease	77 (12.0%)	40 (10.5%)	0.471		
Biliary cancer with distal biliary stricture	58 (9.1%)	30 (7.9%)	0.566		
Pancreatic cancer with distal biliary stricture	96 (15.0%)	92 (24.2%)	< 0.001	1.49 (0.85–2.61)	0.165
Other malignant disease	176 (27.5%)	121 (31.8%)	0.136		
**Post Billroth I reconstruction**	13 (2.0%)	14 (3.7%)	0.111		
**Long oral protrusion**	264 (41.2%)	239 (62.9%)	< 0.001	2.38 (1.80–3.15)	< 0.001
**Peripapillary diverticulum**	127 (19.8%)	75 (19.7%)	0.990		
**Tumor invasion to the papilla or oral protrusion**	25 (3.9%)	31 (8.2%)	0.004	1.61 (0.85–3.06)	0.147
**Distal biliary stricture**	206 (32.1%)	151 (39.7%)	0.014	0.79 (0.54–1.17)	0.238
**Main pancreatic duct stricture**	92 (14.4%)	90 (23.7%)	< 0.001	1.12 (0.64–1.96)	0.687
**Starting operation by trainee**	450 (70.2%)	280 (73.7%)	0.234		

*CI* confidence interval, *ERCP* endoscopic retrograde cholangiopancreatography.

Although 71.5% of the starters were trainees, there was no significant difference in the frequency of requiring salvage techniques compared to expert starters. Trainees performed DGT in 139/179 cases (77.7%), and TPPP in 22/145 cases (15.2%). Only 2/114 cases of NKP were performed by trainees, whereas most were performed by experts. We had to be very careful in allowing trainees to perform NKP, because NKP was freehand incision, which required extensive experience in interventional ERCP^16,18^.

### Length of the oral protrusion

**Figure 4:**
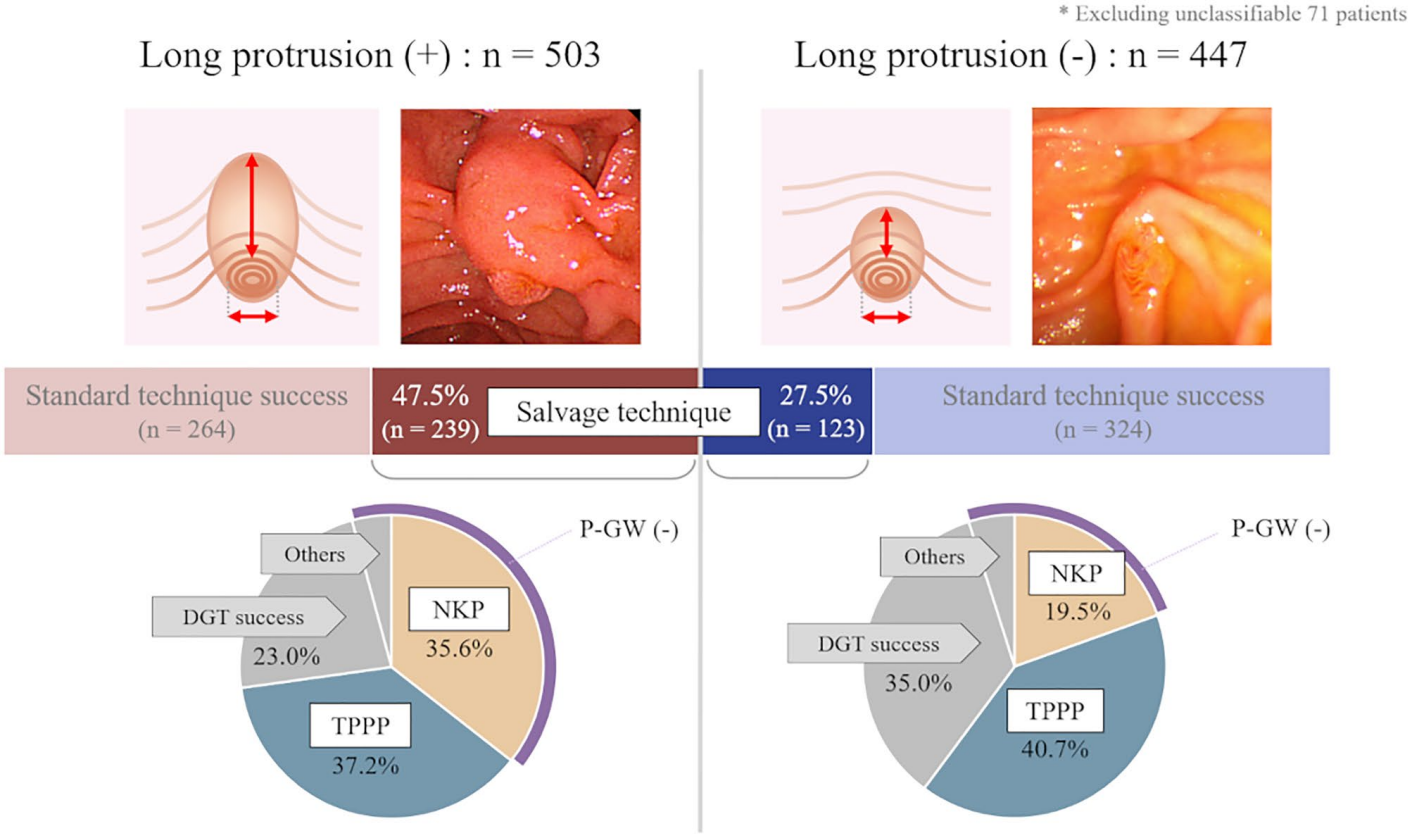
Comparison of cannulation techniques by the length of the oral protrusion. The oral protrusion pattern is classified into two types, according to the length of the oral protrusion. We defined “Long” as more than double the ratio of the length of the oral protrusion to the transverse diameter of the papilla. Salvage techniques are more often required in patients with long oral protrusions than in those without long oral protrusions (66.0% vs. 34.0%, p < 0.001). In such patients, the frequency of NKP was higher because pancreatic guidewire placement was less common. *NKP* needle knife pre-cut, *TPPP* transpancreatic pre-cut papillotomy, *DGT* double-guidewire technique, *P-GW* pancreatic guidewire.

There were 503 cases of long protrusion (LP), and the salvage technique was required in 47.5% of participants in the LP group and 27.5% of participants in the non-LP group (p < 0.001, Fig. 4). In the LP group, the frequency of NKP was higher than in the non-LP group (p = 0.001). After excluding 71 patients who could not be classified due to inadequate pictures, there was no significant difference between the LP and non-LP groups in the success rate of biliary cannulation and the success rates of each salvage technique (DGT, TPPP, and NKP).

## Discussion

In this study, we analyzed the clinical outcomes of the entire process of ERCP from the frontal view of the papilla to selective biliary cannulation in 1021 patients with native papillae. The purpose was to obtain practical knowledge about which salvage techniques should be applied to which patients by reviewing the results of each salvage technique, the choice of those techniques, and the factors that make biliary cannulation difficult.

The strength of this study is that it aggregates more than 1000 cases from a high-volume center and provides a comprehensive analysis of a series of treatment procedures for all subjects without limiting the diseases or techniques. As our institution was a tertiary referral center, many patients were referred after sphincterotomy to make differential diagnosis of biliary stricture or to manage difficult bile duct stones. Therefore, the proportion of cases with native papillae was relatively small. However, the present study would be valuable in that a large number of native papillae treated with a certain strategy was accumulated in a tertiary referral center.

Many studies have reported the results of individual salvage techniques such as pre-cuts or compared 2 or 3 techniques in a limited small number of patients^5,11–20^. There are also several studies analyzing the difficult factors of biliary cannulation^21–25^. On the other hand, no reports have clearly shown the stream of the procedure based on a particular strategy, which cases were difficult, and which salvage techniques were used successfully or not in all the patients. For example, a simple comparison between TPPP and NKP would be exposed to significant bias caused by the difference in the premise of whether pancreatic guidewire could be placed. The study of the overall view of the procedure is important in clinical practice. Our report provides a more clinical perspective on the possibilities and limitations of transpapillary biliary cannulation than many previous reports. To the best of our knowledge, this study is the first report of a large cohort to evaluate the treatment strategy itself in a teaching institution.

This study found that more than half of the patients with long oral protrusions required salvage techniques. Watanabe et al. also defined the Protrusion-L group as the cases in which the length of the oral protrusion was more than twice the transverse diameter of the papilla^27^. They reported that there were significantly more cases of difficult biliary cannulation in the Protrusion-L group. However, their study differed in that the number of patients in the Protrusion-L group was only 61 (10.4% of all patients), and the pre-cut was NKP in all cases, with no mention of TPPP. Although Haraldsson's 4 types classification is also known^28^, we believe that long or not long oral protrusion is a simpler and easier classification and very useful in that it is easy for anyone to evaluate in clinical practice. Our results showed that the rate of unintentional pancreatic cannulation was significantly lower in patients with long oral protrusions than those without; thus, NKP was more frequently chosen as the salvage technique than DGT or TPPP. The long oral protrusion indicates that the bile duct travels a long distance in the duodenal papilla, which means the narrow distal segment (NDS) is long. Therefore, biliary cannulation is considered complicated due to factors such as misalignment of the catheter with the bile duct axis and difficulty in preceding guidewire over the curved NDS. It may also indicate a malignant tumor near the papilla, such as pancreatic ductal carcinoma.

Our institution’s success rate of biliary cannulation in the initial ERCP was 94.3%, and the eventual success rate was 98.3%. The adverse event rate was 4.5% for PEP and 3.2% for bleeding. These results are comparable to those reported by Lee et al., evaluating the ERCP outcomes of 1067 consecutive patients with native papillae^29^. Salvage techniques were required in 380 patients, of which 275 were pre-cut, accounting for 26.9% of the total. This was a higher percentage than that reported by Peng et al.^21^ and Enochsson et al.^30^, both less than 10%. This might be due to the characteristics of our institution, such as the large number of difficult cases referred and the large number of malignant diseases.

Based on our findings, we propose the following strategies when the standard technique is unsuccessful. Regardless of the oral protrusion length, DGT or TPPP should be considered first if a guidewire can be placed in the pancreatic duct and NKP if not. Particularly, in patients with long oral protrusions, a technique change should be actively considered without spending more time than necessary on the standard technique. In such patients, the selection of salvage techniques depends on the existence of a pancreatic guidewire; however, NKP might be a better choice except in patients with easy access to the pancreatic duct. When either TPPP or NKP is ineffective, we can perform the conversion therapy by combining these pre-cut techniques. After several days, a second ERCP could also be a good option in terms of preventing complica events due to prolonged operation time.

Such a strategy should be needed in terms of a safe training program because the training of endoscopists is one of the most important roles of a high-volume center. In our institution, 71.5% of the starters were trainees, and there was no significant difference in the success rate and adverse event rate compared with expert starters. Furthermore, the overall results were not inferior to previous reports that were limited to procedures performed by experts^29^. These are probably due to the well-established system of experts appropriately supervising trainees and taking over procedures as necessary based on a certain strategy.

In recent years, endoscopic ultrasound sonography (EUS) has become widespread. It has been used in biliary and pancreatic diseases in which a transpapillary approach is not possible through standard techniques. A study by Gupta et al. showed that EUS-guided drainage reduced the need for PTBD and surgical procedures^31^. However, EUS-guided drainage has been performed only in a few specialized centers due to cost and technical issues. In areas where ERCP has only recently become a common procedure, the introduction of EUS might still be in the distant future. Thus, in most parts of the world, biliary access is by either a transpapillary or a percutaneous approach. The present study results indicate that a transpapillary procedure can be completed in most cases with a supervising endoscopist who has mastered salvage techniques and an appropriate strategy.

There are some limitations to this study. First, it was a retrospective review of data with its inherent biases. The selection of salvage techniques was made according to a certain strategy, but it depends in part on the judgment based on the experience of the endoscopist, thus it is difficult to determine the optimal selection of salvage techniques in this study. However, a prospective study does not include poor understanding patients or critically ill patients who require urgent treatment, making comprehensive analysis difficult. Second, it is a single-center study. On the other hand, it could be considered a good point to validate a unified ERCP strategy in our institution. Third, several factors may also influence the difficulty of biliary cannulation other than we listed in this study, such as the location of the papillae (e.g., closer to the 3rd position), the depth and speed of breathing, and the strength of intestinal peristalsis. Although it was difficult to collect these precise data retrospectively in this study, further study including these factors would be needed.

In conclusion, this study demonstrated that salvage techniques are useful for patients who have undergone failed standard techniques. Although the salvage techniques were frequently required in patients with long oral protrusions, the success rate of biliary cannulation was high. The safety was excellent under appropriate supervision by an expert. Learning salvage techniques and appropriate selection may help to overcome many difficulties, including cases of initial ERCP failure.

## Supplementary Information

Supplementary Legends.

Supplementary Figure 1.

Supplementary Legends.

Supplementary Figure 2.

## Abbreviations

ERCP: Endoscopic retrograde cholangiopancreatography
DGT: Double guidewire technique
NKP: Needle knife pre-cutting
TPPP: Transpancreatic pre-cut papillotomy
OR: Odds ratio
CI: Confidence interval
PTBD: Percutaneous transhepatic biliary drainage
PEP: Post-ERCP pancreatitis
IQR: Inter-quartile range
EUS: Endoscopic ultrasonography
