# Supplementary material for: “Salvage techniques” are the key to overcome difficult biliary cannulation in endoscopic retrograde cholangiopancreatography
Source: Sci Rep. 2022 Aug 10;12:13627. doi: 10.1038/s41598-022-17809-5 (PMC9365799; doi:10.1038/s41598-022-17809-5)
Supplement: Supplementary file 1 — Supplementary Legends. [file 41598_2022_17809_MOESM1_ESM.docx]

**Supplementary figure S1.** Flow chart of this study.

ERCP, endoscopic retrograde cholangiopancreatography.
