# Supplementary figures and images for: “Salvage techniques” are the key to overcome difficult biliary cannulation in endoscopic retrograde cholangiopancreatography
Source: Sci Rep. 2022 Aug 10;12:13627. doi: 10.1038/s41598-022-17809-5 (PMC9365799; doi:10.1038/s41598-022-17809-5)

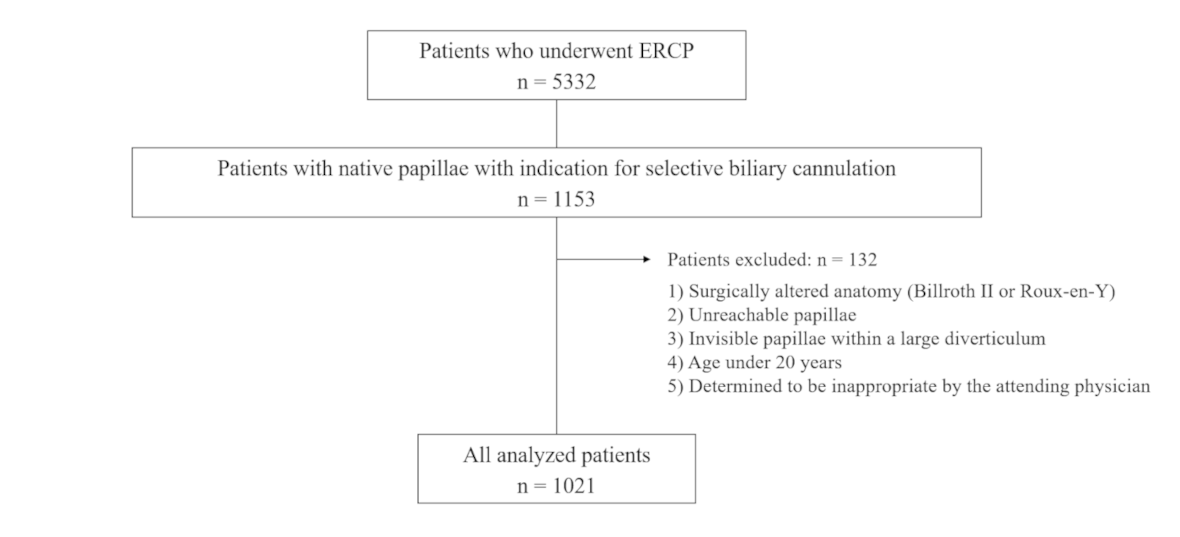

Supplement: Supplementary file 2 — Supplementary Figure 1. [file 41598_2022_17809_MOESM2_ESM.tif]

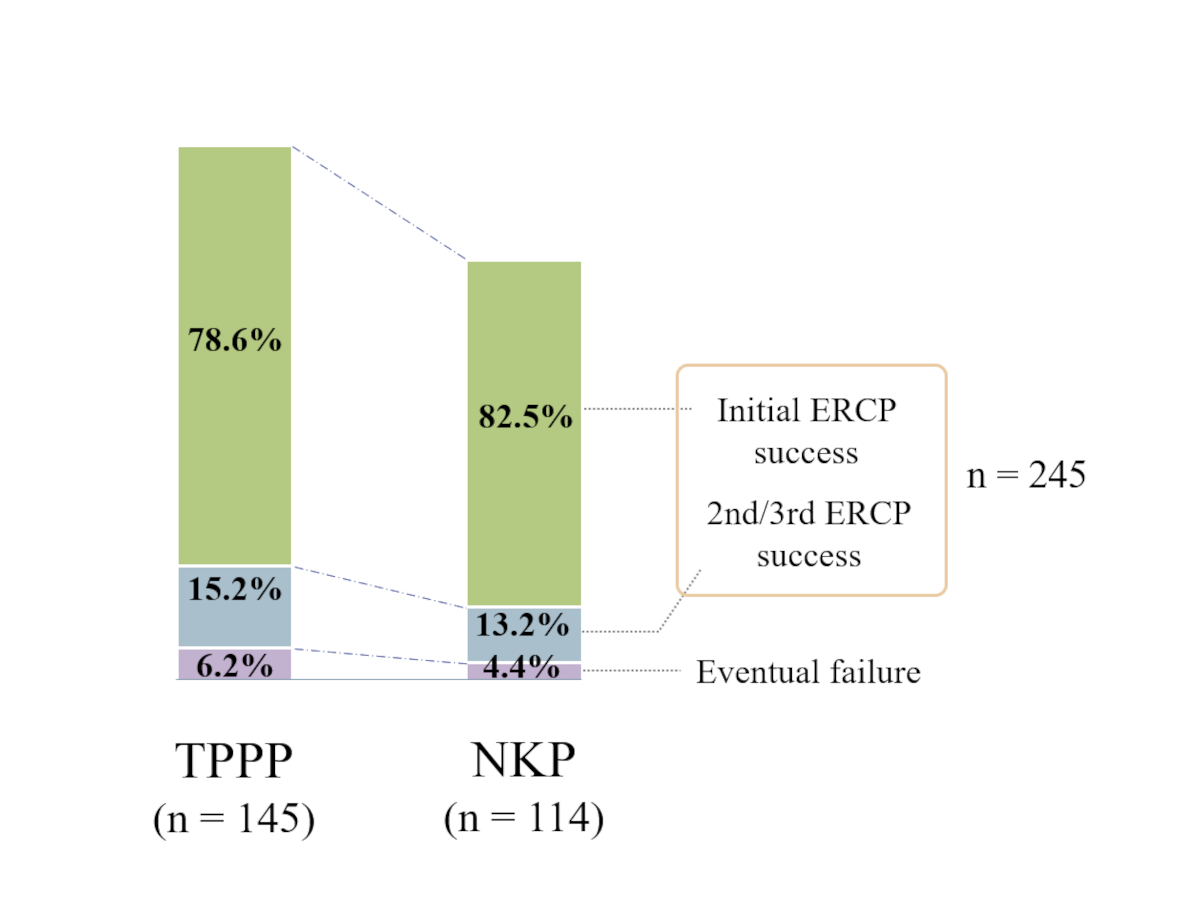

Supplement: Supplementary file 4 — Supplementary Figure 2. [file 41598_2022_17809_MOESM4_ESM.tif]
