# Supplementary material for: “Salvage techniques” are the key to overcome difficult biliary cannulation in endoscopic retrograde cholangiopancreatography
Source: Sci Rep. 2022 Aug 10;12:13627. doi: 10.1038/s41598-022-17809-5 (PMC9365799; doi:10.1038/s41598-022-17809-5)
Supplement: Supplementary file 3 — Supplementary Legends. [file 41598_2022_17809_MOESM3_ESM.docx]

**Supplementary figure S2.** Success rates of each of the primary pre-cut techniques.

There was no significant difference in success rate between the TPPP group and the NKP group (93.8% vs. 95.6%, respectively, p = 0.590). The number of successfully cannulated patients with pre-cut was 245, which was approximately a quarter of the total number of successes.

ERCP, endoscopic retrograde cholangiopancreatography; NKP, needle knife pre-cut; TPPP, transpancreatic pre-cut papillotomy.
